# Memory in the Neonate Brain

**DOI:** 10.1371/journal.pone.0027497

**Published:** 2011-11-07

**Authors:** Silvia Benavides-Varela, David M. Gómez, Francesco Macagno, Ricardo A. H. Bion, Isabelle Peretz, Jacques Mehler

**Affiliations:** 1 Cognitive Neuroscience Sector, International School for Advanced Studies (SISSA/ISAS), Trieste, Italy; 2 Center for Advanced Research in Education (CIAE), University of Chile, Santiago, Chile; 3 Department of Neonatal Pathology, Santa Maria della Misericordia Hospital, Udine, Italy; 4 Department of Psychology, Stanford University, Stanford, California, United States of America; 5 BRAMS, Université de Montréal, Montréal, Canada; University of Otago, New Zealand

## Abstract

**Background:**

The capacity to memorize speech sounds is crucial for language acquisition. Newborn human infants can discriminate phonetic contrasts and extract rhythm, prosodic information, and simple regularities from speech. Yet, there is scarce evidence that infants can recognize common words from the surrounding language before four months of age.

**Methodology/Principal Findings:**

We studied one hundred and twelve 1-5 day-old infants, using functional near-infrared spectroscopy (fNIRS). We found that newborns tested with a novel bisyllabic word show greater hemodynamic brain response than newborns tested with a familiar bisyllabic word. We showed that newborns recognize the familiar word after two minutes of silence or after hearing music, but not after hearing a different word.

**Conclusions/Significance:**

The data show that retroactive interference is an important cause of forgetting in the early stages of language acquisition. Moreover, because neonates forget words in the presence of some –but not all– sounds, the results indicate that the interference phenomenon that causes forgetting is selective.

## Introduction

Immediately after birth, infants are surrounded by a myriad of new sounds. For the first time, the newborn brain has access to enough acoustic detail to distinguish all words in the surrounding language. Yet, questions related to the parts of speech that can be remembered and the mechanisms that constrain this capacity have not been investigated in depth. Can the human brain remember words heard moments after birth? If so, is the representation of these words resistant to interfering sounds?

Numerous studies indicate that neonates are sensitive to acoustic properties of speech and can recognize familiar sounds. Neonates discriminate between rhythmically different languages [1]-[3], and they distinguish all phonetic contrasts in the world's languages [4]-[8]. Moreover, neonates prefer a story heard during the last weeks of pregnancy to a new story [9], and their native language to a foreign language [10]. However, it is unlikely that fetuses remember details about the sound forms of words (hereafter referred to as *word* or *words*), because the properties of the uterus render many phonemic differences imperceptible [11]. There is some evidence that newborns retain a word over a brief delay [12], and even over a day [13], but it is not clear how newborns succeeded in these experiments given that no other study has demonstrated that infants remember common words from the surrounding language before four months of age [14].

In the present study, we used functional near-infrared spectroscopy (fNIRS) to investigate neonates' ability to remember words. Crucially and differently from previous studies, we focused on the causes of forgetting in early infancy. We hypothesized that forgetting of words is higher when the initial encoding event is followed by similar auditory experiences, creating interference between the past and present.

**Figure 1:**
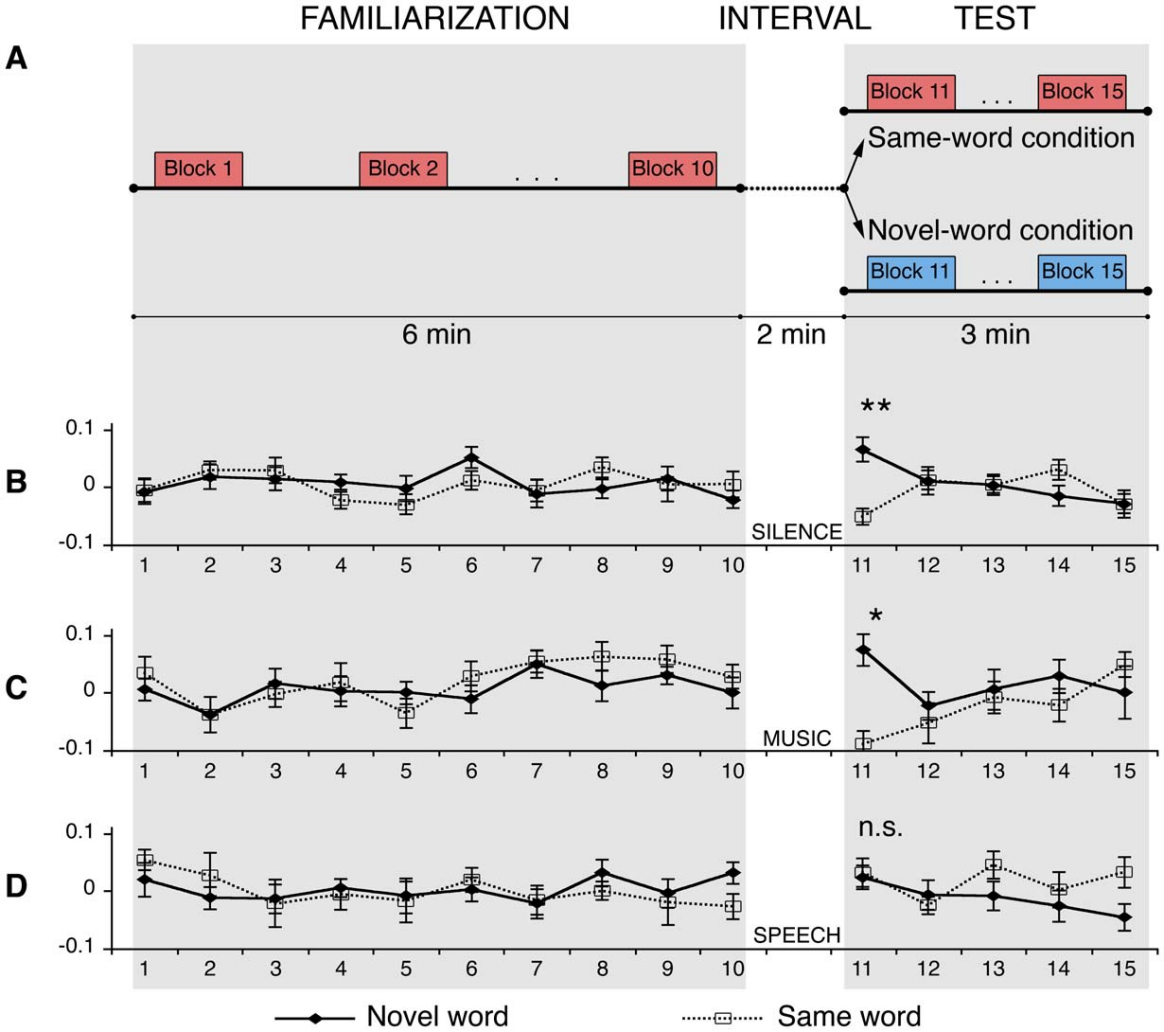
Experimental paradigm and results. **A.** Schematic diagrams of the procedure used in the experiments. During the familiarization phase, all the neonates were presented with 10 blocks composed of 6 identical words. A period of silence of varying duration (from 25s to 35s) followed each block. In the test, 5 blocks of the same word heard during familiarization were presented to half of the neonates while the other half heard a novel word. In Study 1, a silent 2-minute interval intervened between familiarization and test. In studies 2 and 3, the silent interval was filled by music (Study 2) or speech stimuli (Study 3). **B-C-D)** Time courses of the relative hemodynamic changes averaged across all the channels and subjects per group. The dashed line indicates the time series for the group that heard the same word before and after the pause; the continued line represents the group that heard a novel word in the test. Error bars indicate standard errors. The x-axis shows number of blocks; in the y-axis the changes in concentration of Oxy-hemoglobin in mmol*mm is displayed. The neonates who heard a novel word after a silent period showed greater cortical Oxy-Hb concentration changes in the test than neonates who heard the same word before and after the silent pause. The presence of speech stimuli during the interval affects recognition memory in neonates. No interference was found when music was presented during the interval (*, *p*<0.01; **, *p*<0.0001).

In order to test this hypothesis, we familiarized newborns to a word, and then incorporated different auditory stimuli between the end of the familiarization period and the onset of the test, hereafter referred to as the *retention interval* (see **Fig. 1A**). A silent retention interval was our baseline condition to assess memory in the newborn brain. In order to explore forgetting, we used a novel word or instrumental music as interfering stimuli. Differences in the amount of interference caused by a novel word and by music can give insights into how stimuli in these two auditory domains are represented in the newborn brain. Speech and music have different neural encodings in the adult brain [15]-[16], are acoustically distinct, and are assumed to be common in the neonate surroundings.

Based on interference theories [17], we pose that if neonates represent speech and music alike, both stimuli should equally impair word recognition. Alternatively, if newborns represent words and instrumental music differently, verbal information should cause greater interference in word recognition than music.

While previous studies on memory in newborns relied on behavioral responses, we looked at brain responses instead. fNIRS is a non-invasive brain imaging technique that measures hemodynamic responses in the cerebral cortex without requiring any overt behavioral response. This property facilitates the observation of abilities that might have been undetected in previous behavioral investigations. Recently, several laboratories have successfully used fNIRS to test precocious auditory competences in newborns and young infants [18]-[24].

## Results

### Study 1: Word recognition after silent intervals

In our first experiment, we used fNIRS to track the functional hemodynamic responses of the newborn brain when encoding a consonant-vowel-consonant-vowel (CVCV) word. Neonates were familiarized with a nonce word (e.g., *mita*), and after a 2 min silent interval (**Fig. 1A**), they were presented either with the same word heard during familiarization (e.g., *mita*, Same-word condition, also referred to as familiar word) or a novel CVCV word (e.g., *pelu*, Novel-word condition). Notice that the introduction of the 2 min interval before the test phase differentiates the present work from previous discrimination studies in which the test immediately follows the habituation. The familiar and novel words were recorded by the same speaker and had the same syllabic structure, stress pattern, duration and intensity (see methods section). The novel and familiar words were counterbalanced across participants, and no differences in activation were found during familiarization to the word *mita* or *pelu* (permutation tests, all *ps*>0.30, see methods section), showing that any difference in the hemodynamic responses in the test cannot be interpreted merely as a response to the stimulus. Fifty-six neonates were included in the analysis.

Results from the test phase indicated that newborns recognized the familiar word, suggesting that they encoded enough acoustic detail to distinguish it from the novel word after a silent retention interval (**Fig. 1B**). Significant differences in brain activation between the Same-word and Novel-word conditions were observed in the first block of the test (permutation tests, *p*<0.0001 for oxyHb; p<0.01 for deoxyHb). This was the only block in which significant differences in brain activity between neonates in the Novel-word and in the Same-word conditions were found, in either the familiarization or test phases. In addition, differences in the responses from the first block of the test and the last block of the familiarization showed a decrement of oxyHb in the Same-word condition and an increment in the Novel-word condition (permutation tests, *p*<0.01). The concentration of deoxyHb showed a decrement in the Novel-word condition and an increment in the Same-word condition (permutation tests, *p*<0.01).

**Figure 2:**
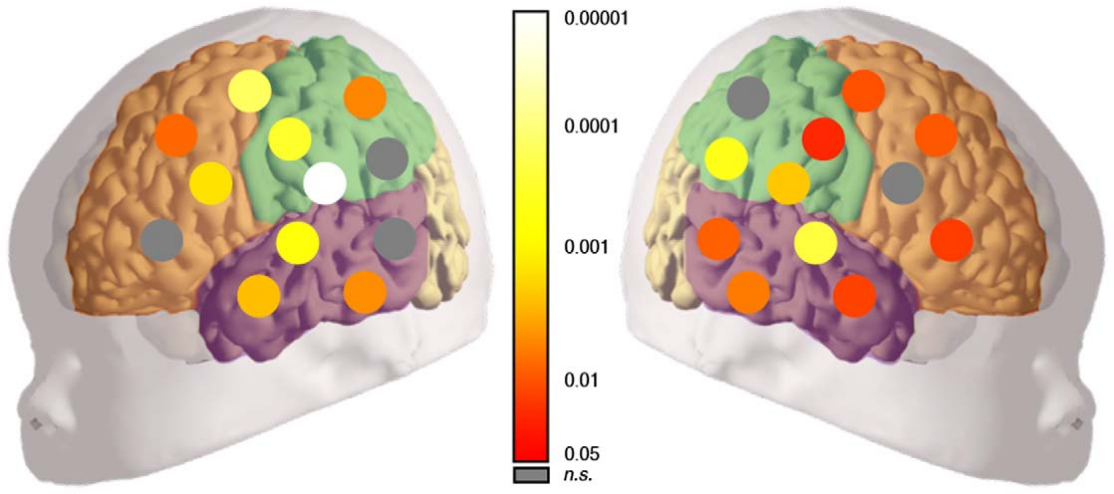
Statistical maps on the schematic neonate brain. The graph depicts the comparison between the Novel-word and Same-word conditions in the first block of the test -block 11, Study 1-. Significance levels for each channel (p-values corrected by false discovery rate [38]) are color-coded as indicated on the color bar. Grey circles indicate no significant differences between conditions.

**Figure 3:**
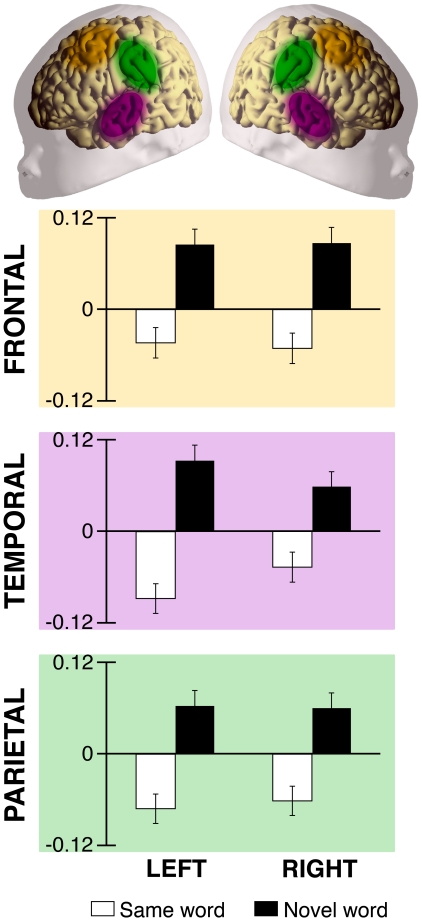
OxyHb changes from the last familiarization block to the first test block (Study 1). Channels bilaterally located in frontal, temporal and parietal areas show a decrease in the concentration of oxyHb when neonates hear the same word before and after the pause (white bar). In contrast, when neonates are confronted with a novel word in the test (black bar) the concentration from the familiarization phase to the test increases. Colored ellipses on the schematic neonate brain indicate the localization of the channels included in the areas of interest.

In order to determine the brain areas that contributed to the overall difference found in the test phase, we compared with t-tests the activation elicited by the two conditions during the first test block in each of the 24 recording points (**Fig. 2**). In addition, we compared brain responses –as measured by oxyHb changes– in six regions of interest: frontal, temporal, and parietal regions of the left and right hemispheres (**Fig. 3**). We observed a main effect of Condition [ANOVA, F(1,54) = 18.469; *p*<0.0001] due to the overall difference in brain activation for neonates in the Novel-word condition as compared to neonates in the Same-word condition. There were no main effects of Hemisphere [ANOVA, F(1,54) = 1.231; n.s.] or Area [ANOVA, F(2,108) = 1.154; n.s.], and no significant interactions between factors [all *Fs*<1]. Our data show that word recognition evokes a diffuse cortical response in the neonate brain, which is bilaterally spread over temporal, parietal, and frontal areas. Because fNIRS is not suitable to measure changes in deeper brain areas, whether or not the observed pattern of activation is partially responsible for word recognition should be clarified in future studies.

These results show that the newborn brain is able to encode a word from brief exposure. Exposure for over half an hour -or over two hundred repetitions of the words- was used in previous studies looking at memory in newborns [12], [13]. The familiarization phase of our experiment was much shorter (six minutes in total, including more than four minutes of silent pauses) and with fewer instances of the familiarization word, providing evidence that newborns do not require protracted experience to remember a word.

The question arises as to the level of detail that the newborns remembered. Did they retain a holistic representation of the word? Were some syllables or phonemes better encoded than others? Was only some information about the word or its syllables (onset, nucleus) encoded? Whereas in the current study, the familiar and the novel words had entirely different sets of phonemes, future studies with words sharing only some syllables or phonemes will be able to establish the detail with which the newborn brain encodes speech stimuli. Still, our study provides some first insights about the information newborns store when they hear words. Neonates maintain the representation of a CVCV word (or parts of it) over the silent retention interval and compare this representation with the sound of a new CVCV word presented during test. Importantly, the two words are pronounced by the same speaker and have similar acoustic properties, suggesting that the information encoded by newborns goes beyond low-level perceptual features such as pitch, voice quality, duration, or intensity.

### Study 2: Word recognition after intervening melodies

Despite the recognition capacity demonstrated in Study 1, newborns' early experiences take place in surroundings that are very different from the silent environment used in our study. Thus, in studies 2 and 3, we investigated whether newborns' memory for words could withstand interfering auditory stimuli.

In Study 2, a new group of neonates (divided again in Same-word and Novel-word conditions) encountered new auditory stimuli during the retention interval. In this study, an excerpt of instrumental music was played between familiarization and test. Under these circumstances, a recognition response would demonstrate that neonates were able to remember a word, and could overcome the interference from music (see details in the method section).

**Figure 4:**
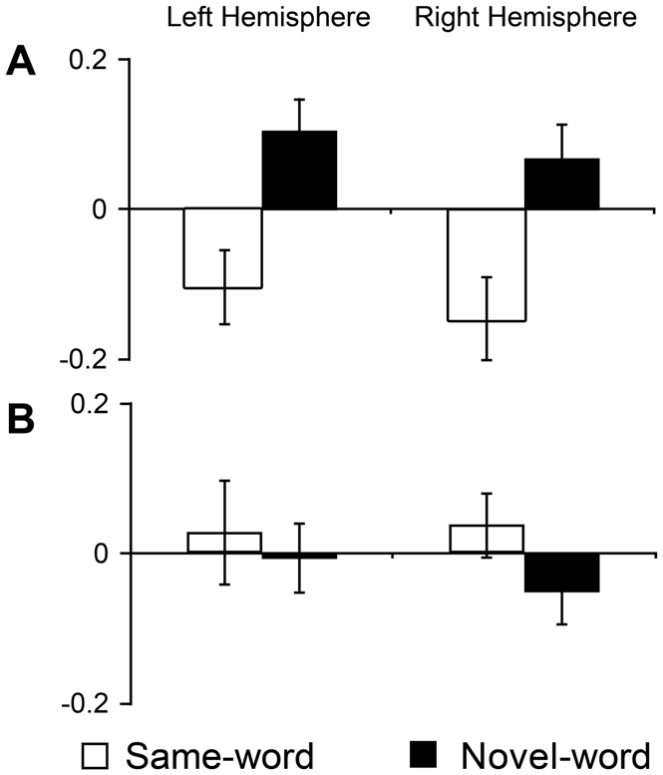
OxyHb changes from the last familiarization block to the first test block (Studies 2 and 3). **A.** When the 2-minute pause was filled with music, channels in both hemispheres showed a decrement in the concentration of oxyHb from the familiarization to the test in the Same-word condition, and an increment in the Novel-word condition. **B.** There were no significant changes in oxyHb concentration from the familiarization to the test phases when the interval was filled with speech stimuli.

Our results show that newborns remembered the new word despite the intervening melody (**Fig. 1C**). As in Study 1, significant differences in hemodynamic activity between conditions were found exclusively in the first block of the test phase; participants in the Novel-word condition showed greater concentrations of oxyHb than participants of the Same-word condition (permutation tests, *p*<0.01). A distributed network including temporo-parietal and frontal areas was again responsible for the observed difference. Furthermore, changes in oxyHb concentration between the last block of the familiarization and the first block of the test differed between the two conditions (permutation tests, *p*<0.05). Participants in the Same-word condition showed a decrease in concentration of oxyHb between familiarization and test, while participants in the Novel-word condition displayed increased hemodynamic responses (**Fig. 4A**). The oxyHb concentration in the first block of the test was significantly different between conditions (ANOVA, main effect of Condition [F(1,26) = 19.318; *p*<0.0001]). There was no main effect of Hemisphere [ANOVA, F(1,26) = 0.058; n.s.], Area [ANOVA, F(2,52) = 0.011; n.s.], and no significant interactions between factors [all Fs<1]. As in the previous study, participants assigned to the Same-word and Novel-word conditions did not differ during the familiarization phase.

These results confirm that newborns are able to recognize a familiar word after a retention interval of a few minutes. In addition, this study shows that newborns represented the word in a format that can resist interference from music.

However, these results do not necessarily imply that this word representation would resist interference from more similar auditory stimuli. In fact, previous studies with adults have suggested that auditory stimuli suffer from highly specific interference effects (e.g. listening to tones interferes with the memory of previously heard tones, but listening to digits does not, [25]). Therefore, in Study 3 we asked whether newborns represented the word in a format that could resist interference from another word.

### Study 3: Word recognition after intervening speech

In Study 3 we tested a new group of newborns with a paradigm identical to the one used in Study 2, except that we presented the word *noke* (instead of instrumental music) during the retention interval. In this study, we hypothesized that the speech stimuli would cause greater interference than the music used in Study 1, possibly causing newborns to forget the word heard during familiarization. This result would suggest additionally that words and music are processed differently in the newborn brain.

In contrast with our previous results, we found no evidence that newborns recognized the familiarization word. We found no significant differences between the conditions in the test phase (**Fig. 1D**). We also failed to observe significant variations in oxyHb-concentration between the last familiarization block and the first block of the test phase (permutation test, *p*>0.50) (**Fig. 4B**). Together these results suggest that the presence of another word in a portion of the retention interval disrupted newborns' ability to recognize the previously heard word. As in the previous studies, we found no significant differences during the familiarization phase.

## Discussion

In laboratory studies, newborns can retain the sounds of words [12], [13], but there is no evidence in the literature showing that infants younger than 4 months of age can remember words from their surrounding language. In this work, we found hemodynamic responses correlated with word recognition in the neonate brain. Our studies also suggest possible causes of forgetting in very young infants. In Study 1, we showed that newborns familiarized with a bisyllabic word distinguished it from a novel word. Brain responses measured with fNIRS revealed a significant recognition response when familiarization and test phases were separated by a 2-minutes silent retention interval.

Studies 2 and 3 focused on the role of interference on forgetting. Study 2 demonstrates that the neonate brain recognizes a word after exposure to instrumental music, whereas Study 3 shows that this memory trace is diminished when a different word is presented before the test. As we hypothesized, newborns' memory for words is affected when it is followed by similar auditory stimuli.

An interesting area for speculation constitutes the level over which the newborn brain computes similarity between speech and music. What counts as similar auditory stimuli for newborns? As a first alternative, it is possible that similarity is computed with reference to low-level acoustic cues. The word presented during the familiarization and retention interval had the same pitch, duration, intensity, voice quality, syllabic structure, and stress pattern. In contrast, the instrumental music had a more complex melodic contour and continuous transitions. This alternative predicts that it is the degree of similarity –acoustic, but not phonemic– between the familiarization word and the intervening word that determines the amount of interference. A second alternative is that similarity might be computed with reference to the source that produced the auditory stimuli. Words are generated by the vocal tract, while instrumental melodies are produced by artifacts. This alternative predicts that a humming melody produced by the vocal tract would interfere with the memory for words. As a third alternative, similarity between music and speech might be computed at abstract levels that are processed by specialized brain mechanisms. The second and third alternatives are supported by studies showing that the neonate brain is specialized to process speech [22], [26]. Additional support for these alternatives comes from studies showing that speech and music are treated differently in the human brain [15]-[16], [27]-[28], and that vocal and non-vocal sounds are processed by different brain areas [22], [29]-[30].

Newborns are able to remember a word, but how are these words encoded? Linguists and psychologists have proposed that speech is encoded as sequences of articulatory gestures [31]-[32], as features, phonemes, syllables, or prosody [33]-[36]. Future studies should focus on the nature of speech encoding at birth. Whatever this encoding might be, it probably does not generalize to music sequences [15]-[16].

Here we provide evidence that humans are able to memorize words hours after birth. Our findings also suggest that interference is one of the causes of forgetting in early infancy. The word and the instrumental music used during the retention interval elicit different processes in the neonate brain. Future studies should investigate whether this differential processing and interference generalizes to a broader variety of speech and melodies, determining the extent to which the human brain is pre-wired to interpret the auditory world.

## Methods

### Participants

Fifty-six neonates (27 females, mean age 3.1 days, range 1-5 days) were included in Study 1. Thirteen additional neonates were tested but excluded from the analysis because of head movements that produced large motion artifacts (n = 7), or because they cried before the end of the experiment (n = 6). In Study 2, 28 healthy full-term neonates (15 females, mean age 2.8 days, range 1-5 days) participated in the experiment. Five neonates were tested but excluded from the analysis because head movements produced large motion artifacts (n = 3), or because of crying before the end of the experiment (n = 2). Finally, in Study 3, 28 new healthy full-term neonates (12 males, mean age 2.9 days, range 1-5 days) were included in the analysis. Eight additional neonates were tested but excluded from the analysis because movements produced large motion artifacts (n = 7), or because of crying before the end of the experiment (n = 1). We failed to obtain signals from neonates who had thick hair. Neonates were recruited at the newborn nursery of Azienda Ospedaliera Universitaria Santa Maria della Misericordia in Udine, Italy. Neonates were considered eligible if they had gestational ages between 38 and 42 weeks, Apgar scores ≥8 in the first minute and diameter of head ≥33.5 cm. Bioethics Committee of SISSA/ISAS (International School for Advanced Studies) approved the study; all parents signed an informed consent before the experiments.

### Stimuli

The three pseudo words used (*mita, pelu, noke*) were pronounced using a neutral intonation, carried first syllable stress, had a CVCV (consonant-vowel-consonant-vowel) structure, and were edited to have the same intensity (70dB) and duration (700 ms). An adapted excerpt of a Brahms' waltz played on a piano was used in Study 2. Its duration was 10 s (the same length of a block composed of 6 words) and had a mean intensity of 70dB.

### Procedure

The experiment consisted of a familiarization phase, an interval, and a test phase (**Fig. 1A**). The familiarization phase lasted six minutes and was organized in ten blocks. Each block contained six identical words. Within blocks, words were separated by pauses of randomized length (0.5 s or 1.5 s), yielding blocks of approximately 10 s each. Blocks were separated by time intervals of varying duration (25 s or 35 s). A 2-minute interval was inserted between the end of the familiarization and the beginning of the test phase. The interval in Study 1 was a silent interval. In Study 2, thirty seconds were occupied by three blocks of Brahms' lullaby. In Study 3, this interval was occupied by three blocks of another word. The test consisted of five blocks (3 minutes). Neonates were tested while lying in their cribs, asleep or in a state of quiet rest. A nurse assisted neonates inside a dimly lit sound-attenuated booth where the experiment was run. Sound stimuli were presented via two loudspeakers placed at a distance of 1.2 m from the infant's head at a 30° angle on both sides, raised to the same height as the crib. The speakers were connected to a Macintosh power PC G5 computer that at the same time operated the NIRS machine and presented the auditory stimuli using PsyScope X software (http://psy.ck.sissa.it/). Both the NIRS machine and the computer were placed outside the experimental booth and were controlled by the experimenter. An infrared video camera was used to monitor the infant's behavior.

For half of the participants in each experiment, the stimulus of the familiarization phase was *mita* and the novel word in the test was *pelu*. For the other half of participants, the words were exchanged, so that *pelu* was used during the familiarization phase and *mita* was presented as the novel word in the test. All analyses used pooled data from all newborns, since there was no significant difference between participants in any of the blocks (permutation test, all *ps*>0.30), evidencing that the acoustic properties of the words *per se* are not responsible for different neural responses.

### Data acquisition

A NIRS machine (ETG-4000, Hitachi Medical Corporation, Tokyo, Japan) was used. The separation between emitters and detectors was 3 cm and the sampling rate 10 Hz. The total laser power output per fiber was 0.75 mW and the two continuous light sources used 695 nm and 830 nm wavelengths. Probes holding the fibers were placed on the neonate's head by using skull landmarks. We obtained simultaneous recordings from 24 points (channels). Although individual variation cannot be excluded, placement maximizes the likelihood of monitoring the temporal, parietal and frontal areas. Channels from 1 to 12 were placed on the left hemisphere and from 13 to 24 on the right hemisphere. Channels 1, 2, 4 and 5 were roughly located in the left frontal regions; 3, 6, 8 and 11 in the left temporal and 7, 9, 10 and 12 in the left parietal region. Channels 13, 14, 15 and 16 were in the right frontal area; Channels 17, 19, 22 and 24 in the right temporal and 18, 20, 21 and 23 in the right parietal region.

### Data Processing and Analysis

Our analysis was based on the variations in oxy-hemoglobin (oxyHb) concentrations assessed on the basis of the light absorption recorded by the NIRS machine. The signal was band-pass filtered between 0.02 Hz and 1.00 Hz to remove components arising from slow fluctuations of cerebral blood flow, heartbeat and other possible artifacts. Single blocks from specific channels were eliminated on the basis of two criteria: 1) light absorption of less than 1% of the total light emitted (generally because the probes were not touching the neonate's scalp); 2) the presence of large movement artifacts. The criterion to detect artifacts was the presence of rapid changes in the signal (>0.1 mmol*mm in an interval of 0.2 s). Blocks with more than 12 rejected channels were excluded. Participants were included in the analysis only if the amount of data rejected was less than 30%.

For the non-rejected blocks, a baseline trend was linearly fitted between the mean of the 5 s preceding the onset of the block and the mean of the 5 s between the 25^th^s and the 30^th^s after the onset of the block. For each channel, the mean signal changes in the period between the end of the auditory stimulation and the following 9s (where the maximum amplitudes of the hemodynamic responses are expected) were used to carry out the subsequent statistical analysis.

To assess whether the two conditions differed across the familiarization phase, we computed the maximum difference between the mean activation for each condition. That is to say, for each block b = 1,2,…, we calculated
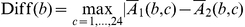



where 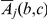 is the activation in block b and channel c, averaged among all neonates assigned to condition j (Same-word/Novel-word). When analyzing the familiarization phase as a whole, we further computed Diff(fam) as the maximum of Diff(b) for all blocks b = 1,2,…,10. Because the distribution of this statistic is not Gaussian, we evaluated significance using non-parametric methods. Specifically, we used permutation tests [37]. In these tests, a distribution for the test statistic under the null hypothesis is obtained by re-randomizing the condition assigned to each subject. Assuming that the two conditions do not differ during the familiarization phase, then the distribution of Diff(fam) is the same for the original group assignment as for any random reassignment. Significance is then computed as the proportion of reassignments exhibiting a value of Diff(fam) greater or equal than the one associated to the original groups. Other statistics such as Diff(test) are built and statistically evaluated in the same way. We used 10,000 random reassignments for each permutation test.

Additional tests were conducted to identify the channels contributing to the differences in the test phase that the previous analysis found. We compared the two groups on a channel-by-channel basis using 2-sample t-tests. To solve the problem of multiple comparisons, we computed corrected p-values based on the procedure proposed in [38; Theorem 1.3] to control the False Discovery Rate at the 5% level. That is, starting from the 24 uncorrected p-values (one per channel) *p*_(1)_, *p*_(2)_, …, *p*_(24)_ sorted from smallest to largest, corrected p-values were obtained as 
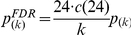
, with c(24) defined as 1+1/2+1/3+…+1/24.

To examine larger brain areas associated with auditory memory, we compared activation during the last block of the familiarization phase in frontal, temporal and parietal areas. We used a repeated-measures analysis of variance (ANOVA) with Condition (Same-word/Novel-word) as a between-subject factor and Area (frontal/temporal/parietal) and Hemisphere (left/right) as within-subject factors. Two channels were included in each area-hemisphere region. In the left frontal, channels 2 and 5; left temporal, 3 and 6; left parietal, 7 and 9; right frontal, 13 and 15; right temporal, 17 and 19; and right parietal, 18 and 21. These channels were chosen based on previous imaging studies of auditory processing in neonates and young infants [19].
